# BMC Urology reviewer acknowledgement 2014

**DOI:** 10.1186/1471-2490-15-1

**Published:** 2015-01-26

**Authors:** Hayley Henderson

**Affiliations:** BioMed Central, Floor 6, 236 Gray’s Inn Road, London, WC1X 8HB UK

## Abstract

The editors of *BMC Urology* would like to thank all our reviewers who have contributed to the journal in Volume 14 (2014).

**Mehdi Abbasi**

Iran

**Ibrahim Abdel-Hamid**

Egypt

**Jan Adamowicz**

Poland

**Mayank Agarwal**

India

**Madhu Agrawal**

India

**Diego Aguilar**

Ecuador

**Shabbir Alibhai**

Canada

**Satoshi Anai**

Japan

**Jonathan Aning**

UK

**M. S. Ansari**

India

**Fernando Antonanzas**

Spain

**Abdullah Armagan**

Turkey

**Andrew Armstrong**

USA

**Mohammad Asim**

UK

**Jae Hyun Bae**

Korea, South

**Yong-Rui Bai**

China

**Mirko Bertozzi**

Italy

**Bimal Bhindi**

Canada

**Daniele Bianchi**

Italy

**Antonella Biroli**

Italy

**Kari Bø**

Norway

**Sharon Bober**

USA

**Katharina Boehm**

Germany

**Michael Boettcher**

Germany

**Fadi Brimo**

Canada

**Willem Brinkman**

Netherlands

**Stefano Bucci**

Italy

**Alessandro Calarco**

Italy

**Alberto Calori**

Italy

**Estrella Carballido**

USA

**Rufus Cartwright**

UK

**Valéria Carvalho**

Brazil

**Pietro Castellan**

Italy

**Fausto Catena**

Italy

**Francesco Celestino**

Italy

**Ravi Chandra**

USA

**Ming-Yuan Chen**

China

**Jun Chen**

China

**Sreenivasulu Chintala**

USA

**Kian Chong**

Singapore

**Bilal Chughtai**

USA

**Felix Chun**

Germany

**Antonio Cicione**

Italy

**Luca Cindolo**

Italy

**Peter Clark**

USA

**Andrea Cocci**

Italy

**Luigi Cormio**

Italy

**Simone Crivellaro**

USA

**Jican Dai**

China

**Seth Davis**

Canada

**Piergustavo De Francesco**

Italy

**Simona Di Francesco**

Italy

**Giuseppe Di Lorenzo**

Italy

**Fernando Domingos**

Portugal

**Ross Drayton**

UK

**Blythe Durbin**

USA

**Michael Eisenberg**

USA

**Faysal Ekici**

Turkey

**Shawky Elhaggar**

Egypt

**Ahmed El-Zawahry**

USA

**Bradley Erickson**

USA

**Ali Erol**

Turkey

**Arnold Etame**

USA

**Walid Farhat**

Canada

**Enrico Finazzi Agro**

Italy

**Josef Finsterer**

Austria

**Davide Firinu**

Italy

**Mayer Fishman**

USA

**John Fitzgerald**

USA

**Jay Fowke**

USA

**Rodrigo Fraga-Silva**

Switzerland

**Frank Friedersdorff**

Germany

**Michael Froehner**

Germany

**Hhroyuki Fujimoto**

Japan

**Kiyohide Fujimoto**

Japan

**Yasuhito Funahashi**

Japan

**Chunkit Fung**

USA

**Maria Furlan**

Italy

**Riccardo Galli**

Italy

**Fabrizio Gallo**

Italy

**John Gearhart**

UK

**Petrisor Geavlete**

Romania

**Ahmed Ghaith**

Egypt

**Guido Giusti**

Italy

**Marc Goldstein**

USA

**Dragan Golijanin**

USA

**Alex Gomelsky**

USA

**Umut Gönülalan**

Turkey

**Cemil Göya**

Turkey

**Marco Grande**

Italy

**Sezgin Günes**

Turkey

**Caj Haglund**

Finland

**Robert G Hahn**

Sweden

**Oliver Hakenberg**

Germany

**Jin Han**

Korea, South

**Jens Hansen**

UK

**Michael Hanzly**

USA

**Reina Haque**

USA

**Nicholas Harding-Jackson**

USA

**Ashraf Hassan**

Egypt

**Hannelore Heemers**

USA

**Elmar Heinrich**

Germany

**Thomas Hermanns**

Switzerland

**Thomas Reinhard William Herrmann**

Germany

**Lars Holmberg**

Sweden

**Hisashi Honjo**

Japan

**Lee Hugar**

USA

**Valerio Iacovelli**

Italy

**Kenneth Iczkowski**

USA

**Jonathan Ilicki**

Sweden

**Manuela Ingrosso**

Italy

**Takahiro Inoue**

Japan

**Hendrik Isbarn**

Germany

**Joseph Ischia**

Australia

**Mustafa Okan Istanbulluoglu**

Turkey

**Paulo Jaworski**

USA

**Tony John**

USA

**Lu Jun**

China

**Klaus-Peter Jünemann**

Germany

**Takashi Karashima**

Japan

**Wassim Kassouf**

Canada

**Martin Kathrins**

USA

**Tomoyuki Kato**

Japan

**Takashi Kawahara**

Japan

**Mitsuhiro Kawano**

Japan

**Mohamed Keheila**

Egypt

**Hosni Khairy**

Egypt

**N Khandelwal**

India

**Wael Y Khoder**

Germany

**Dae Kim**

USA

**Soodong Kim**

Korea, South

**Won Tae Kim**

Korea, South

**Zachary Klaassen**

USA

**Thomas Kolon**

USA

**Chuize Kong**

China

**Evan Kovac**

Canada

**Hann-Chorng Kuo**

Taiwan

**Masaomi Kuwada**

Japan

**Danny Landau**

USA

**Jean-Baptiste Lattouf**

Canada

**Nathan Lawrentschuk**

Australia

**Souhil Lebdai**

France

**Seung Wook Lee**

Korea, South

**Una Lee**

USA

**Michael Leitzmann**

USA

**Sara Lenherr**

USA

**Costantino Leonardo**

Italy

**Sandrine Leroy**

France

**Xiaomeng Li**

China

**Vincenzo Li Marzi**

Italy

**Ching-Chung Liang**

Taiwan

**Sey Kiat Lim**

Singapore

**Hsueh-Kung Lin**

USA

**Huiyi Lin**

USA

**Wei-Chou Lin**

Taiwan

**Alvin Liu**

USA

**Adam Luchey**

USA

**Lorenzo Luciani**

Italy

**Amit Mahipal**

USA

**Jose Mansure**

Canada

**Eugenio Martorana**

Italy

**Giuseppe Martorana**

Italy

**Eric Massanyi**

USA

**Hitoshi Masuda**

Japan

**Luigi Mearini**

Italy

**Joshua Meeks**

USA

**Jonathan Melquist**

USA

**Florence Menegaux**

France

**Jesse Mills**

USA

**Majid Mirzazadeh**

USA

**Makito Miyake**

Japan

**Naoto Miyanaga**

Japan

**Juan Moreno**

Spain

**Alicia Morgans**

USA

**Jason Muhitch**

USA

**Razvan Multescu**

Romania

**Yasutoshi Murayama**

Japan

**Rosa Nadal**

Spain

**Yasushi Nakai**

Japan

**Hitoshi Nakashima**

Japan

**Nissrine Nakib**

USA

**Shunichi Namiki**

Japan

**Shintaro Narita**

Japan

**Neema Navai**

USA

**Kenneth Nepple**

USA

**Lindsay Nicolle**

Canada

**Richard Norman**

Canada

**Rikke Norregaard**

Denmark

**Takahiro Ochiya**

Japan

**Mi Mi Oh**

South Korea

**Chinonyerem Okoro**

USA

**Ephrem Olweny**

USA

**Takehisa Onishi**

Japan

**Rahmi Onur**

Turkey

**Mehmet Ruhi Onur**

Turkey

**Yen-Chuan Ou**

Taiwan

**Ian Pagano**

USA

**Giovanni Palleschi**

Italy

**Dong Soo Park**

Korea, South

**Hyun Jun Park**

Korea, South

**Bo Parys**

United States Minor Outlying Islands

**Electra Paskett**

USA

**Antonio Luigi Pastore**

Italy

**Kathryn Penney**

USA

**Dennis Peppas**

USA

**Gianpaolo Perletti**

Italy

**Zoran Persec**

Croatia

**Maria Picken**

USA

**Eugene Pietzak**

USA

**Geraldine Pignot**

France

**Giovannalberto Pini**

Sweden

**Rui Pinto**

Portugal

**Giacomo Maria Pirola**

Italy

**Zenon Pogorelic**

Croatia

**Frederic Pouliot**

Canada

**Mariela Pow Sang**

Peru

**Ahmet Kursad Poyraz**

Turkey

**Fabrizio Presicce**

Italy

**Silvia Proietti**

Italy

**Stefano Puliatti**

Italy

**Richard Quinton**

UK

**Maria Theresa Redaniel**

UK

**Zaccaria Ricci**

Italy

**Benjamin Ristau**

USA

**Alejandro Rodriguez**

USA

**Marco Rosa**

Italy

**Charles Rosser**

USA

**Jason Rothwax**

USA

**Giorgio Ivan Russo**

Italy

**Amanda Sacker**

UK

**Hooman Sadri-Ardekani**

USA

**Amirali Salmasi**

USA

**Francesco Sanguedolce**

UK

**Richard Santucci**

USA

**Christos Savopoulos**

Greece

**Helmut Schiffl**

Germany

**Peter Schraml**

Switzerland

**Thomas Schwaab**

USA

**Thomas Sebo**

USA

**Wade Sexton**

USA

**Jay Shah**

USA

**Ali Shamsa**

Iran

**Ahmad Shamsodini**

Qatar

**Qiang Shao**

China

**Pranav Sharma**

USA

**Bobby Shayegan**

Canada

**Katsumi Shigemura**

Japan

**Sungryul Shim**

Korea, South

**Brian Shuch**

USA

**Prabhjot Singh**

India

**Zachary Smith**

USA

**Petros Sountoulides**

Greece

**Preston Sprenkle**

USA

**Giorgio Stanta**

Italy

**Robert Stoehr**

Germany

**Kelly Stratton**

USA

**Ramnath Subramaniam**

UK

**Ruth Tachezy**

Czech Republic

**Mostafa Taymour**

Egypt

**Angelo Territo**

Italy

**Atsushi Tomioka**

Japan

**Roser Torra**

Spain

**Filippo Maria Turri**

Italy

**Santiago Vallasciani**

Italy

**Stephen Van Den Eeden**

USA

**Marry Van Den Heuvel-Eibrink**

Netherlands

**E.P. Van Haarst**

Netherlands

**Jill Vanmassenhove**

Belgium

**Krishnan Venkatesan**

USA

**Andrew Vickers**

USA

**Matteo Vittori**

Italy

**Anna Walker**

UK

**Ming Wang**

USA

**Wolfgang Weidner**

Germany

**Heinric Williams**

USA

**Jan Wohlfahrt**

Denmark

**Addie Wootten**

Australia

**Zhang Xiansheng**

China

**Faysal Yafi**

Canada

**Stanley Yap**

USA

**Danny Youlden**

Australia

**Ezekiel Young**

USA

**Kamran Zargar Shoshtari**

New Zealand

**Qiusheng Zheng**

China

**Jian Zhuo**

China

**Alexandre Zlotta**

Canada

**Alessio Zordani**

Italy

**Ellen Zwarthoff**

Netherlands

**Debra Zynger**

USA

